# Hydraulic conductance, resistance, and resilience: how leaves of a tropical epiphyte respond to drought

**DOI:** 10.1002/ajb2.1323

**Published:** 2019-07-11

**Authors:** Gretchen B. North, Erin K. Brinton, Marvin G. Browne, Madeline G. Gillman, Adam B. Roddy, Tiffany L. Kho, Emily Wang, Vitor A. Fung, Craig R. Brodersen

**Affiliations:** ^1^ Department of Biology Occidental College Los Angeles CA USA; ^2^ Department of Ecology and Evolutionary Biology University of California Los Angeles CA USA; ^3^ School of Forestry & Environmental Studies Yale University New Haven CT USA; ^4^ Biology Department University of San Francisco San Francisco CA USA; ^5^ Department of Biotechnology Johns Hopkins University Baltimore MD USA

**Keywords:** aerenchyma, aquaporins, Bromeliaceae, cuticular conductance, embolism, *Guzmania monostachia*, intercellular air space, leaf anatomy, tank bromeliad

## Abstract

**Premise:**

Because of its broad range in the neotropical rainforest and within tree canopies, the tank bromeliad *Guzmania monostachia* was investigated as a model of how varying leaf hydraulic conductance (*K*_leaf_) could help plants resist and recover from episodic drought. The two pathways of *K*_leaf_, inside and outside the xylem, were also examined to determine the sites and causes of major hydraulic resistances within the leaf.

**Methods:**

We measured leaf hydraulic conductance for plants in the field and laboratory under wet, dry, and rewetted conditions and applied physiological, anatomical, and gene expression analysis with modeling to investigate changes in *K*_leaf_.

**Results:**

After 7 d with no rain in the field or 14 days with no water in the glasshouse, *K*_leaf_ decreased by 50% yet increased to hydrated values within 4 d of tank refilling. Staining to detect embolism combined with modeling indicated that changes outside the xylem were of greater importance to *K*_leaf_ than were changes inside the xylem and were associated with changes in intercellular air spaces (aerenchyma), aquaporin expression and inhibition, and cuticular conductance.

**Conclusions:**

Low values for all conductances during drying, particularly in pathways outside the xylem, lead to hydraulic resilience for this species and may also contribute to its broad environmental tolerances.

Epiphytes in the tropical rainforest face an uncertain future, in large measure because the present warming and drying trends are likely to continue (Phillips et al., 2010). Because of their potential exposure to high levels of sunlight and wind and varying levels of rainfall, tropical epiphytes have been described as unusually vulnerable in a changing climate (Colwell et al., 2008; Zotz, 2016), particularly those that depend on abundant atmospheric moisture to meet transpirational demand (Zotz and Bader, 2009). Yet some epiphytes with large latitudinal and within‐canopy distributions, such as the subject of this investigation, *Guzmania monostachia*, have been shown to have broad environmental tolerances, particularly with respect to light (Griffiths and Maxwell, 1999). Moreover, in response to different prevailing light conditions, plants of *G. monostachia* differ in the rate at which their leaves move water (North et al., 2016). The adaptability of *G. monostachia* under changeable light and water supplies has been attributed at least in part to its expression of CAM activity under stressful conditions (Maxwell et al., 1992, 1994). Whether epiphytes will be at the leading edge of tropical plant mortality as droughts increase in frequency and duration may depend on the flexibility of several traits, including leaf hydraulic conductance, and the coordination of such traits in conferring resilience.

In tropical rainforests, epiphytes typically experience annual rainfall in excess of 1000 mm (Gentry and Dodson, 1987), yet water availability can be uncertain on a daily and seasonal basis for plants lacking root systems in soil. Consequently, vascular epiphytes are characterized by a number of adaptations that prolong their supply of water, including absorptive leaf trichomes, water storage tissue in leaves (e.g., hydrenchyma) and roots (e.g., velamen), and extra‐plant water storage in overlapping leaf bases (tanks) (Benzing, 2008; Zotz, 2016). Moreover, epiphytes that occupy exposed sites in the upper tree canopy also tend to possess water‐conserving traits such as CAM photosynthesis, low rates of stomatal conductance, and low rates of cuticular water loss (Kerstiens, 1996; Helbsing et al., 2000). In addition, the generally low growth rates of vascular epiphytes (Zotz, 2016) can help plants persist through unfavorable conditions, including drought.

Because of the wide geographic and spatial distribution of the tank bromeliad *G. monostachia*, its variable leaf hydraulic conductance (*K*_leaf_) in different light environments (North et al., 2016), and the likelihood of future changes in rainfall in much of its range, we consider *G. monostachia* as a useful model for drought‐induced plasticity in the pathways of water movement within a leaf. We measured *K*_leaf_ for plants exposed to drying and rewetting in the field in Costa Rica and in a glasshouse. Mature plants of *G. monostachia* are predicted to be able to tolerate and recover from 12 days with no water in their tanks (Zotz and Thomas, 1999); thus, our first hypothesis was that *K*_leaf_ for plants in the glasshouse would decrease during 2 weeks with dry tanks yet would be at least partially restored by rewetting. We tested this hypothesis in the field in Costa Rica and at the end of a naturally occurring dry period.

Recent work on leaf hydraulics under drying conditions has focused on possible changes in the balance between conductance through the xylem, *K*_x_, and outside the xylem, *K*_ox_ (Trifiló et al., 2016; Scoffoni et al., 2017). Previous studies that have sought to compare the relative contributions of *K*_x_ and *K*_ox_ have used various methods, such as measuring *K*_leaf_ before and after cutting leaf veins or lamina tissue to remove resistances and subtracting the component resistance from the whole leaf resistance (Wei et al., 1999; Sack et al., 2005; Trifiló et al., 2016). A few studies have used values of *K*_x_ calculated from xylem dimensions together with measured values of *K*_leaf_ and Ohm's analogy or other models to calculate *K*_ox_ (Martre et al., 2001; Xiong et al., 2017), while others have modeled *K*_ox_ directly from detailed anatomical measurements and physical variables involved in water transport (Buckley, 2015; Scoffoni et al., 2017). In the current study, *K*_leaf_ was measured using the evaporative flux method (Sack et al., 2002), *K*_x_ was calculated from tracheid diameters, and a “leaky cable” model of water transport as modified for leaves was used to calculate *K*_ox_ (North et al., 2013, 2016). Like most members of the Bromeliaceae, leaves of *G. monostachia* possess only tracheids as conducting elements in the xylem (Tomlinson, 1969; Males, 2016).

Because the relative contributions of the two conductance pathways in leaves of *G. monostachia* differ depending on the light environment (North et al., 2016), we investigated whether drought would also affect the two pathways differentially. Thus, our second hypothesis was that changes in *K*_leaf_ would involve changes in the xylem pathway (*K*_x_) due to drought‐induced embolism, which we investigated by whole‐leaf stain uptake. Our third hypothesis was that drought‐induced decreases in *K*_ox_ would occur alongside a number of changes in extravascular traits such as intercellular air space, vein spacing, leaf thickness, and cuticular conductance, as well as in the expression of aquaporins, membrane‐spanning proteins that are essential in water transport in leaf tissues outside the xylem (Nardini et al., 2005; Sade et al., 2014). Our approach was to apply combined anatomical, physiological, and modelling methods to help distinguish potential sources of vulnerability or resilience in leaf water transport for *G. monostachia* during drought.

## MATERIALS AND METHODS

### Plant material from La Selva, Costa Rica

*Guzmania monostachia* (L.) Rusby ex Mezis is a tank bromeliad that occurs naturally from Brazil to Florida and at several different heights in the tree canopy (Griffiths and Maxwell, 1999). Field measurements were made on plants of *G. monostachia* in a wet, lowland tropical forest managed by the Organization for Tropical Studies, La Selva Biological Station (84°00′12″W, 10°25′52″N) in northeastern Costa Rica. Leaf hydraulic conductance (*K*_leaf_) was measured for five mature plants growing in full sun on a tree trunk in a forest clearing at the end of the dry season (June 2016), when the tanks were empty for at least 7 d (dry treatment). Four days after rainfall, when the tanks were full, leaves were collected from the same five plants (rewetted treatment). For each plant, the fourth or fifth leaf from the center of the rosette was cut at the base with a razor blade, placed in a black plastic bag with wet paper toweling, and taken to a climate‐controlled laboratory where leaf bases were recut under water, immersed in distilled water, and allowed to rehydrate overnight. *K*_leaf_ was measured the next morning using the same methods as in the laboratory in Los Angeles, described below.

For measuring cuticular conductance (*g*_min_), leaves were collected from five mature plants growing in or near forest clearings, bagged, and brought to an ambient lab, with continuously monitored but unregulated conditions of temperature and relative humidity. Leaves were kept in darkness for 3–4 h and then sealed at the cut base of the lamina and on the abaxial (stomatous) side (the adaxial side of *Guzmania* leaves lack stomata; Tomlinson, 1969; North et al., 2013) with two applications of spray‐on liquid bandage to reduce possible stomatal conductance. Further detailed procedures for measuring and calculating *g*_min_ for plants in La Selva and glasshouse‐grown plants are described below.

### Plants in glasshouse conditions, Los Angeles, CA

Plants of *G. monostachia* were purchased from a nursery in Florida (Michael's Bromeliads, Venice, FL, USA). At the end of experimentation, plants were allowed to flower to confirm species identification. Plants for measurements of *K*_leaf_, *g*_min_, and associated anatomical and morphological traits under controlled wet, dry, and rewetted conditions were grown in a shaded glasshouse in Los Angeles, CA, USA (34°7′39″N, 118°12′37″W) for at least 30 d before experiments were begun. Light levels in the glasshouse averaged 20% of ambient solar radiation (with a maximum PAR of ca. 360 μmol m^−2^ s^−1^); daily average maximum/minimum temperatures were ca. 30.5/21.5°C. For the eight plants in wet conditions, tanks (cupped leaf bases) were initially filled to capacity with a dilute commercial nutrient solution for bromeliads and subsequently kept filled with deionized water. The same eight plants were then subjected to dry conditions by emptying the tanks with a pipette and withholding water for 14 d. For rewetted conditions, the tanks of the plants previously subjected to dry conditions were refilled to capacity with deionized water.

### Leaf hydraulic conductance (*K*_leaf_)

Leaf hydraulic conductance, *K*_leaf_ (m^3^ m^−2^ s^−1^ MPa^−1^ or mmol m^−2^ s^−1^ MPa^−1^) was measured using the evaporative flux method (Sack et al., 2002). The fourth or fifth leaf from the center of the plant was removed, and its base was immersed in distilled water previously filtered (pore, size 0.2 μm) and degassed by spinning under a vacuum. The leaf lamina (blade) was then recut with a razor blade directly above the tank region; because the tank region was not included, *K*_leaf_ was measured for the leaf blade only, with water entry through the xylem exposed by cutting the leaf base. The leaf was placed in a vial of degassed water that covered approximately 5 mm at the base of blade; plastic film was used to secure the leaf in the vial and prevent evaporation. The vial was placed on a 0.1 mg balance, and its mass was recorded every 10 s. Although *G. monostachia* can shift to CAM photosynthesis during drought, measurable stomatal conductance has not often been observed after dark but instead tends to be highest in the early morning (Maxwell et al., 1992, 1994); thus, *K*_leaf_ was measured under lights in the morning. The leaf was illuminated by red and blue LED lighting that produced 500 μmol m^−2^ s^−1^ of PAR at the top of the leaf, and a small fan kept air moving to eliminate boundary layers. Leaf temperature averaged 23°C, and air and water temperature averaged 20–22°C (water temperature values were normalized to 20°C).

When mass readings stabilized, usually within 10 min, mass was recorded and graphed for 30 min, and the slope of the line was used to calculate volumetric flow (*Q*, m^3^ s^−1^), which is the equivalent of leaf transpiration, *E*, when expressed on a leaf area basis (mmol m^−2^ s^−1^). After the leaf was removed from the balance, it was bagged for 5 min and its water potential *Ψ*_leaf_ (MPa) was measured with a pressure chamber (PMS Instruments, Portland, OR, USA). Leaf blade length, width, and area were determined from digital photographs, using the program ImageJ (Schneider et al., 2012). Leaf hydraulic conductance, *K*_leaf_, was calculated from the average slope of leaf transpiration divided by *Ψ*_leaf_:

$$K_{\mathrm{leaf}}=\frac{E}{{-\Psi }_{\mathrm{leaf}}}$$

### Leaf axial hydraulic conductance (*K*_x_): xylem measurements

The maximum theoretical axial xylem hydraulic conductance (*K*_x_, m^4^ s^−1^ MPa^−1^) was calculated based on xylem anatomy because aerenchyma channels running longitudinally through the leaf blade prevented direct pressure‐driven measurement of water flow. Freehand cross‐sections of leaves from plants used in measurements of *K*_leaf_ were made with a razor blade. Sections were stained with 0.1% w/v toluidine blue O in phosphate buffer and photographed at magnifications of 40–1000× using a Spot RT Color digital camera (Diagnostic Instruments, Sterling Heights, MI, USA) mounted on a Nikon Eclipse ME 600 light microscope (Nikon, Melville, NY). Using ImageJ with calibrated photographic images, the number of veins per millimeter was measured for leaf cross sections viewed at 40× and multiplied by the leaf width to determine the number of main veins across the leaf. Only main veins, which in *G. monostachia* are parallel and in one rank, were measured; secondary lateral veins were short and relatively infrequent and were not considered in the calculation of *K*_x_. Tracheid diameters in at least 20 veins were measured per leaf, treating tracheids as circles with diameter equal to that of the largest circle to be circumscribed within the tracheid; this treatment may underestimate the true theoretical conductance (Lewis and Boose, 1995). Calculating theoretical *K*_x_ in this way, however, tends to overestimate actual xylem conductance because it does not take into account variables such as pit membrane resistance, tracheid taper (Schulte et al., 1987; Choat et al., 2008), and inter‐conduit connectivity, which may become more limiting if conduits become embolized (Jacobsen and Pratt, 2018; Mrad et al., 2018). The diameters *d* (m) were used in the Hagen–Poiseuille equation to calculate *K*_x_ (Nobel, 2009):

$$K_{x}=\sum_{i=1}^{N}\frac{\pi d_{i}^{4}}{128\eta },$$

where *N* is the number of tracheids in each vein multiplied by the number of veins in the leaf, and *η* is the viscosity of water (values normalized to 20°C).

### Staining to detect and estimate embolism

To distinguish between conducting and non‐conducting veins as an estimate of embolism, leaves from plants used in *K*_leaf_ measurements were recut under water at the base of the blade under water, and the cut end was quickly immersed in tracer dye. Four stains were tried, safranin O, phloxine, basic fuchsin, and acid fuchsin, each dissolved in filtered, degassed water. Acid fuchsin, at 0.2% w/w and pH 5.1 (Kitin et al., 2010; Umebayashi et al., 2010), moved more rapidly and evenly than did the other stains, appearing at the tip of the blade within 2 h of application (Appendix S1). Similar results were obtained with dye injection to visualize water transport in *Pieris japonica* (Umebayashi et al., 2007). Perhaps because the solution of acid fuchsin at pH 5.1 was more acidic than the solutions of basic fuchsin (pH 5.7), safranin (pH 6.2), and phloxine (7.1), it did not adhere to the negatively charged walls of the tracheids and thus served as the best mimic of water movement in the xylem (Umebayashi et al., 2007). The leaf was kept for 2 h in the dye near a fan under LED lighting, and then several 5‐mm^2^ sections were immediately cut from the center of the leaf, mounted whole on microslides under water, and photographed at 40×. All stained and unstained main veins were counted, and (number of unstained veins/total number of veins) × 100 was used to approximate percent embolism. Cross sections were examined to verify that acid fuchsin moved through the xylem and stained tracheids, although stain was observed occasionally in bundle sheath cells as well.

### Leaf hydraulic conductance outside the xylem (*K*_ox_)

Conductance of tissues outside the xylem (*K*_ox_, m^3^ m^−2^ s^−1^ MPa^−1^, or mmol m^−2^ s^−1^ MPa^−1^) was calculated using leaf dimensions, measured values for *K*_leaf_, and values for *K*_x_ calculated using the Hagen‐Poiseuille equation in a model based on leaky cable theory originally developed for roots (Landsberg and Fowkes, 1978; Alm and Nobel, 1991) and modified for leaves (North et al., 2013). This model was used in preference to the more common electrical analog model because the two components of leaf hydraulic conductance, xylem conductance (*K*_x_) and conductance outside the xylem (*K*_ox_) do not occur strictly in parallel nor in series (Wei et al., 1999). The model assumes that, along the length of the leaf *l* (m), there is an axial flux through the xylem and a radial flux through tissues outside the xylem, driven by a potential difference between the source of water at the base of the leaf and at the point of evaporation near the leaf surface. In the model,

$$K_{\mathrm{ox}}=\frac{k_{\mathrm{leaf}}\;\alpha l}{\text{tanh}\;\alpha l},$$

where *α* = $\sqrt{\frac{wK_{\mathrm{ox}}}{K_{x}}}$ and *w* = leaf width (m) (North et al., 2013). Initially, *K*_ox_ was set equal to *K*_leaf_ and a solution was obtained by iteration. Units for *K*_leaf_, *K*_x_, and *K*_ox_ were normalized by leaf area and leaf length for the sake of comparison and converted to mmol m^−2^ s^−1^ MPa^−1^. Specifically, to express conductances in comparable units, values of *K*_x_ obtained from the Hagen‐Poiseuille equation (m^4^ s^−1^ MPa^−1^) were divided by leaf length, converted to molar equivalents, and expressed on a leaf area basis, as were *K*_leaf_ and *K*_ox_.

### Leaf anatomy

**Figure 1:**
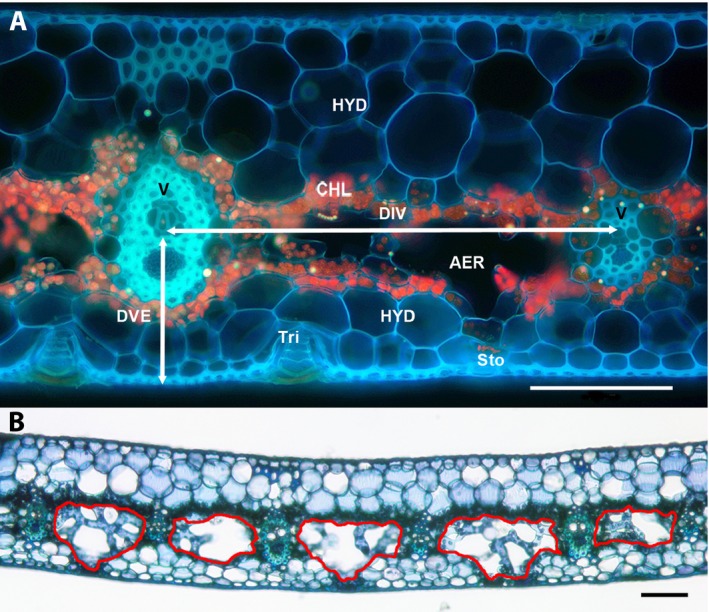
Cross sections of leaves of *Guzmania monostachia* (A) unstained, viewed using UV epifluorescence, labeled with veins (V), bundle sheaths (BS), aerenchyma (AER), chlorenchyma (CHL), hydrenchyma (HYD), distance from vein to abaxial epidermis (DVE), distance between veins (DIV), stomate (Sto), and trichome (Tri); and (B) stained with toluidine blue, with regions of aerenchyma outlined in red; scale bar = 100 μm.

Vein length per area of leaf (VLA, mm mm^−2^, or mm^−1^) was measured on leaves adjacent to those used in measurements of *K*_leaf_, cleared and stained with safranin O (Ruzin, 1999). Four fields per leaf were photographed at 40× (field of view, 7.1 mm^2^), and vein lengths were measured using ImageJ. Other anatomical variables were measured from freehand cross‐sections of leaves used in measurements of *K*_leaf_ that had been fixed in formalin–acetic acid–alcohol (FAA), (Ruzin, 1999), rinsed, and stained with toluidine blue O. Due to concerns about possible shrinkage, comparisons of fixed sections with freshly cut material were examined and showed no significant differences in variables such as leaf thickness and tissue thicknesses (Appendix S2). The distance from the center of main veins to the abaxial epidermis, DVE (μm), was measured from leaf cross sections (Fig. 1A), which were also used to measure the interveinal distance, DIV (μm), the linear distance from the center of one vein to the center of the next. Leaf thicknesses were measured from the same cross sections photographed at 100×.

Relative proportions of leaf tissues were determined in two ways. First, freehand tracings of tissue areas (Fig. 1B) were made using ImageJ from photographs of cross sections of leaves of plants used to measure *K*_leaf_. The aerenchyma (Fig. 1A, B) is the region of the mesophyll with large air spaces, the chlorenchyma comprises the chloroplast‐containing layers in the center of the leaf (excluding mesophyll strands in the aerenchyma), and the hydrenchyma comprises the adaxial and abaxial layers of large cells without chloroplasts. Veins and associated cells (lignified bundle sheaths, Fig. 1A) were excluded from measurement of tissue areas because changes in their areas were minimal due to lignification. Second, the amount of air space in the leaf regions between veins was also quantified on a volumetric basis, because recent work has shown that three‐dimensional (3D) imaging can provide additional measurements that are difficult to obtain with 2D imaging (Théroux‐Rancourt et al., 2017). The 2D and 3D comparisons made by Théroux‐Rancourt et al. (2017) included an analysis of *Guzmania lingulata*, which is similar in leaf form to the study species in the present study. Thus, the 2D and 3D approaches used here were viewed as complementary, particularly given the complex geometry of the stellate mesophyll cells that traverse the aerenchyma that could be difficult to characterize with a 2D approach. Representative leaves from two plants from both wet and dry conditions were detached, double‐bagged in plastic with moist paper towels, shipped overnight to the Advanced Light Source at Lawrence Berkeley National Laboratory (beamline 8.3.2) and imaged using high resolution X‐ray computed microtomography (microCT) within 36 h. Leaves were kept in humid bags until immediately before imaging. For microCT imaging, each leaf was placed in a custom Styrofoam holder, while the remaining portions of the leaf outside of the X‐ray beam were draped in moist paper towels to prevent dehydration. This protocol has been sufficient to prevent dehydration of structures as delicate as excised flowers, and any dehydration during the approximately 15‐min scan time was apparent as blurred images. For each scan, the leaf was rotated 180°, capturing 1025 projection images at 24 keV, yielding a final pixel resolution of 1.28 μm. From the reconstructed tomographic slices, air space total and relative volumes were calculated using the BoneJ plugin for ImageJ based on images that had been adjusted for brightness and contrast and processed by median and bilateral filters. Scans were done at three positions (upper, middle, and lower) and for all longitudinal channels in the leaf (channel defined as the region bounded by the upper and lower epidermis, excluding the veins and bundle sheaths on either side). At least three channels per position per treatment were processed.

To trace the movement of water through extravascular tissues, we carefully detached two leaves from each of six plants from wet and dry conditions, cut them at the base of the blade under water, and placed them in 0.5% w/v sulforhodamine G in water, which moves primarily in the apoplast (Canny, 1986). Leaves were put under lights with a fan and allowed to transpire for 3 h, then sectioned just beyond the dye front that could be detected by eye. Cross sections were cut, mounted in silicon oil to prevent dye diffusion, and viewed using a Leica TCS SP5 confocal microscope (Leica Microsystems, Buffalo Grove, IL, USA); the laser was set for emission wavelengths of 530–550 nm.

### Cuticular conductance (*g*_min_)

Leaves from plants under wet, dry, and rewetted conditions were cut and brought to an ambient laboratory at La Selva or a climate‐controlled laboratory in Los Angeles. To eliminate residual water loss from partially closed stomata, we coated half of the leaves from glasshouse‐grown plants with a thin layer of silicon grease on their abaxial (stomatous) side (Kerstiens, 1996), while half were left uncoated. Air temperature and relative humidity were continuously monitored at the lab bench, and a small fan was used to reduce the leaf boundary layer. Leaves were weighed repeatedly for several hours until the rate of mass loss stabilized at a minimum; *g*_min_ (m s^−1^) was calculated as the rate of mass loss divided by 2× the projected area of the leaf multiplied by the difference in water vapor concentration between the leaf and the air, calculated from leaf temperature, air temperature, and the mean relative humidity (Kerstiens, 1996).

### Aquaporin identification and expression

For glasshouse‐grown plants (*N* ≥ 5), the fourth leaf from the center was removed, and 10‐mm sections from the tank (immersed water‐absorbing region) and leaf blade were excised, placed in DNA/RNA Shield (Zymo Research, Irvine, CA, USA), and stored at 4°C until use. DNA was extracted using the DNeasy Plant Mini Kit (Qiagen, Germantown, MD, USA) following manufacturer's instructions. DNA was eluted in 65 μL elution buffer, and DNA concentration was determined using a Qubit 2.0 Fluorometer with a dsDNA High‐Fidelity DNA Assay kit (Thermo Fisher Scientific, Waltham, MA, USA). DNA (100 ng) was amplified via polymerase chain reaction (PCR) using Taq polymerase (Genesee Scientific, San Diego, CA, USA) in 25 μL reaction volumes following manufacturer's instructions using *TiPIP2α* forward and *Gm DNA* reverse primers (Table 1). This forward primer was chosen based on the high level of expression and responsiveness to drought of *TiPIP2a* in leaves of a closely related bromeliad epiphyte, *Tillandsia ionantha* (Ohrui et al., 2007). Similarly, one aquaporin isoform, PIP2;1, proved the most important in regulating rosette hydraulic conductance in *Arabidopsis thaliana* (Prado et al., 2013). Optimal annealing temperature was identified via gradient PCR. PCR was run on an Eppendorf Mastercycler gradient (Eppendorf, Hamburg, Germany) using the following program: initial denaturation at 95°C for 4 min; 35 cycles of denaturation at 95°C for 30 s, annealing at 58°C for 45 s, and extension at 68°C for 1 min; and a final extension at 72°C for 10 min. PCR products were electrophoretically separated, and the darkest band was isolated with a QIAquick Gel Extraction kit (Qiagen). The final product was ligated into pCR4‐TOPO following the TOPO TA cloning kit manufacturer's instructions (Invitrogen) and inserted into competent *E. coli* DH5α cells. Positive colonies and PCR amplicons were sequenced using Sanger sequencing by Laragen, Inc. (Culver City, CA, USA).

**Table 1:** Oligonucleotides and temperatures used in PCR amplicon Sanger sequencing and qRT‐PCR for *GmPIP* from *Guzmania monostachia*.

Gene	Forward	Reverse	Annealing temp (°C)	Product generated
*TiPIP2α*	5′ GATAATGGTG	5′ GCGATCATGT	60	PCR amplicon for sequencing
	AAGAACGTGGAG 3′	ACAGCAACG 3′		
*GmDNA*	5′ GATAGATGCA	5′ GCGCGTAGAA	63	PCR amplicon for sequencing
	AAGGAGCTGA3′	AAGACACA 3′		
*GmPIP*	5′ CCGGTGGTCA	5′ ATCATGTAC	60	qPCR amplicon
	CATAAATC3′	AGCAACGC3′		
*matK*	5′ GAACTTTGGC	5′ GAAAGAACTT	61.5	qPCR amplicon
	TCGTAAACATAAG 3′	GTTCTTCTTCCG 3′		

Evolutionary analyses were conducted in MEGA6 (Tamura et al., 2013). Amino acid sequences of four *PIP* (plasma membrane integral protein) genes in *T. ionantha* were deduced from Ohrui et al. (2007). Sanger sequence data of the single PCR amplicon for *GmPIP* from *G. monostachia* were used to build a nucleotide consensus sequence (archived at GenBank; MK880047), which was translated to an amino acid sequence and then aligned with the four *T. ionantha PIP* sequences and one pineapple *PIP* downloaded from GenBank (ACB56912.1). The alignment was visualized in GeneDoc (Nicholas and Nicholas, 1997), and the *GmPIP* sequence was uploaded to the standard protein BLAST program of the NCBI database to determine the most closely related gene with a publicly available sequence (Altschul et al., 1990).

Total RNA was extracted from all tissue segments (from fourth leaf from *N* ≥ 5 plants) using the Quick‐RNA MiniPrep kit (Zymo Research). Tissue was finely chopped in a 1:1 v/v ratio of DNA/RNA Shield and RNA lysis buffer. Tubes were centrifuged for 1 min at 14,000 × *g*, then the supernatant was transferred to a Spin‐Away Filter. The remaining steps, including an in‐column DNase I treatment, followed manufacturer's instructions. RNA was eluted in 50 μL of DNase/RNase‐Free water and stored at −80°C until further use. RNA concentration was determined with the Qubit.

Single‐stranded cDNA was synthesized from 0.5 μg RNA using oligo dT (Integrated DNA Technologies, Coralville, IA, USA) as primer and Maxima H Minus reverse transcriptase (Thermo Fisher Scientific) according to the manufacturer's protocol recommended for GC‐rich templates. Quantitative reverse‐transcriptase polymerase chain reaction (qRT‐PCR) analyses of total RNA were performed using gene‐specific primers (Table 1). qRT‐PCR was performed following the qPCR 2X GREEN Master Mix Low Rox manufacturer's protocol (Genesee) in 25 μL reactions containing 25 ng cDNA, 0.1 μM of each primer, 9.5 μL ddH_2_O, and 12.5 μL of 2X Master Mix. qRT‐PCR was run on a 7500 Real Time PCR System (Thermo Fisher Scientific). Data for gene‐specific primers were analyzed relative to the maturase K (*matK*) reference gene (Mirjalili et al., 2011) and normalized to the geographic mean (Remans et al., 2014). Gene‐specific primer efficiencies ranged from 90 to 110%. Significant outliers for the data were removed based on a 0.05 significance level using Grubbs test.

### Measurement of *K*_leaf_ with aquaporin inhibitor

To investigate the physiological role of aquaporins under wet, dry, and rewetted conditions, we measured leaf volumetric water flow (*Q*) as described above for *K*_leaf_ and again after treatment of the leaves with 200 μmol HgCl_2_, which was chosen as an inhibitor after it was shown to be most effective in preliminary experiments with different concentrations of both HgCl_2_ and AgNO_3_ (Niemietz and Tyerman, 2002). Six glasshouse‐grown plants of *G. monostachia* were used to measure *Q* following procedures described above. Under wet conditions, after 14 d with no water, and after rewetting for 4 d, both before and after the same leaf was placed with 5 mm of its base immersed in the HgCl_2_ solution for 15 min under light. Flow rates were compared for leaves before and after HgCl_2_ treatment.

### Statistical analyses

Statistical analyses were performed using SigmaPlot 13.0 (Systat Software, San Jose, CA, USA), applying log‐transformations when needed to normalize data and using the Bonferroni correction for multiple comparisons. Pearson product moment correlations between hydraulic conductances and other leaf traits were calculated using SigmaPlot. Data are reported as means ± 1 SE.

## RESULTS

### Leaf hydraulic conductances and resistances

**Figure 2:**
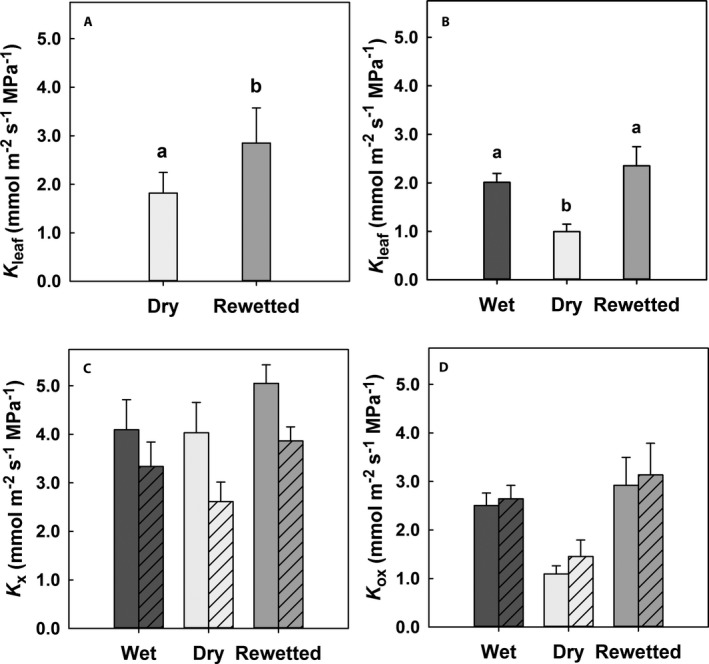
Leaf hydraulic conductance (*K*_leaf_) for leaves of *Guzmania monostachia* (A) in the field in Costa Rica under dry (light gray bars) or rewetted conditions (medium gray bars) and (B) in the glasshouse in Los Angeles under wet (dark gray bars), dry, and rewetted conditions; and hydraulic conductance in the glasshouse (C) through the xylem (*K*_x_) and (D) through the extravascular tissues (*K*_ox_). Bars with diagonals in C and D are conductances calculated using values of *K*_x_ and *K*_ox_ based on the percentage of stained vascular bundles (*K*_x‐stained_ and *K*_ox‐stained_, indicating the effect of embolism). Data are means ± SE for *N* = 5–7 plants; different lower case letters indicate significant differences (*P* < 0.05, one‐way ANOVA).

In the field at La Selva, leaf hydraulic conductance, *K*_leaf_, was measured for *Guzmania monostachia* at the end of the dry season after a month of low rainfall. For five plants that had received no recent rain and had empty tanks for 7 d, *K*_leaf_ was 36% lower than 4 d after rainfall had refilled the tanks (Fig. 2A; *t* = –2.93, df = 4. *P* = 0.043). Leaf water potential, *Ψ*_leaf_, was –0.60 ± –0.04 MPa under dry conditions and –0.24 ± –0.06 MPa after rainfall (*t* = 2.23, df = 10, *P <* 0.001).

In the glasshouse at Los Angeles, a set of seven plants was measured under wet conditions, after 14 d with dry tanks, and after 4 d of rewetting. Leaf hydraulic conductance, *K*_leaf_, decreased by 50% from its value under wet conditions after 14 d under dry conditions and was restored to its initial value by 4 d of rewetting (Fig. 2B; *F*_2, 17_ = 7.00, *P* = 0.006). Under wet and rewetted conditions, *Ψ*_leaf_ did not differ (–0.46 ± –0.02 MPa and –0.42 ± –0.04 MPa, respectively), but *Ψ*_leaf_ was significantly lower under dry conditions (–0.78 ± –0.04; *F*_2, 25_ = 30.25, *P <* 0.001). For a subset of the 7 plants with dry tanks for 30 d, *Ψ*_leaf_ was –0.99 ± –0.26 and *K*_leaf_ was 0.756 ± 0.441 mmol m^−2^ s^−1^ MPa^−1^ (further data not shown; *N* = 3).

**Figure 3:**
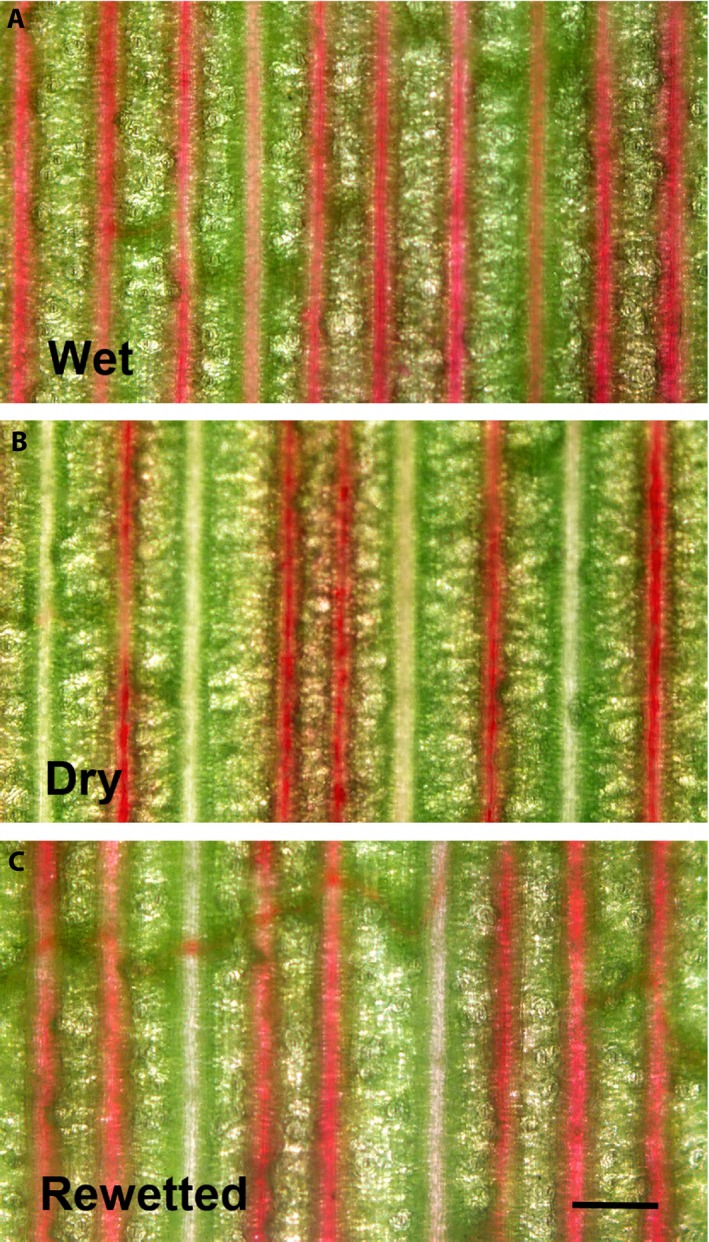
Whole‐leaf segments of *Guzmania monostachia* after leaves were allowed to take up 0.2% w/v acid fuchsin for 2 h to determine which veins were conducting under wet (A), dry (B), and rewetted (C) conditions viewed with a light microscope at 40×. Scale bar = 300 μm.

Theoretical axial hydraulic conductance through the xylem, *K*_x_, as predicted using the Hagen‐Poiseuille equation,  and divided by leaf length and leaf area to make units comparable with *K*_leaf_, did not differ for leaves under the three moisture conditions (Fig. 2C), as expected given that tracheid dimensions and number would not have changed during the 3 weeks of the experiment. Leaves from plants used in measurements of *K*_x_ were allowed to take up acid fuchsin dye for 2 h (Fig. 3), and the percentage of stained veins was 81.5 ± 4.7%, 64.8 ± 2.0%, and 76.5 ± 3.1% under wet, dry, and rewetted conditions, respectively (*F*_7, 2, 14_ = 8.94, *P* = 0.003). Values of *K*_x_ multiplied by these percentages yielded *K*_x‐stained_, an approximation of hydraulic conductance through the xylem decreased by embolism (Fig. 2C). In this regard, leaf cross sections made under the three moisture conditions showed no discernible differences in the shape of tracheids, suggesting that little to no tracheid deformation occurred during drying (Appendix S2). In leaves of a subset of the seven plants with empty tanks for 30 d, 58.9 ± 0.9% of veins were stained (further data not shown; *N* = 3).

Leaf hydraulic conductance outside the xylem, *K*_ox_, calculated using the leaky cable model, was similar to *K*_leaf_, decreasing by 56% during drying and increasing to its initial value after rewetting (Fig. 2D; *F*_2, 26_ = 6.55, *P* = 0.008). Using *K*_x‐stained_ in the model gave *K*_ox‐stained_, which was slightly but not significantly higher than *K*_ox_ (Fig. 2D) simply because *K*_x‐stained_ was lower than *K*_x_.

**Figure 4:**
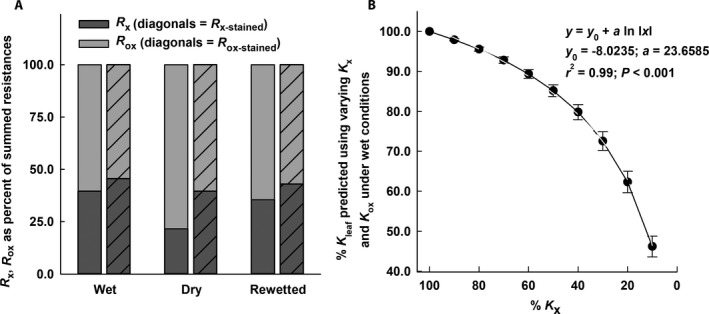
(A) Hydraulic resistances for leaves of *Guzmania monostachia* under wet, dry, and rewetted conditions. Dark gray bars indicate the ratio of resistance through the xylem (*R*_x_, the reciprocal of *K*_x_) to the summed resistances (*R*_x_ plus the resistance of leaf tissues outside the xylem, *R*_ox_); light gray bars indicate the ratio of *R*_ox_ to the summed resistances; bars with diagonals are resistances calculated based on the percentage of stained vascular bundles, indicating the effect of embolism. (B) Percentage of leaf hydraulic conductance (*K*_leaf_) calculated using varying values of *K*_x_ and *K*_ox_ in the leaky cable model.

The two hydraulic pathways, inside and outside the xylem, were expressed as resistances *R*_x_ (1/*K*_x_, normalized by leaf length and area); *R*_x‐stained_ (1/*K*_x‐stained_); *R*_ox_ (1/*K*_ox_); and *R*_ox‐stained_ (1/*K*_ox‐stained_), using values for *K*_ox_ and *K*_ox‐stained_ (from Fig. 2D), generated using the leaky cable model. Resistances were expressed as percentages of their sum to determine which pathway in the leaf was more limiting to leaf hydraulic conductance. Under wet and rewetted conditions, *R*_x_ represented less than 40% of the summed resistances, decreasing to only 22% of the sum under dry conditions (Fig. 4A). When staining was used to indicate reduction in flow due to embolism, *R*_x‐stained_ was 43–45% of the summed resistances under wet and rewetted conditions and 31% of the sum under dry conditions. Under all conditions, even with embolism considered, resistance outside the xylem was more limiting than that inside the xylem; however, because *K*_x_ was calculated using the Hagen–Poiseuille equation and should be considered an ideal or maximum theoretical value, actual xylem resistance is likely to have been higher than reported.

The relative contribution of *K*_x_ was also assessed using the leaky cable model with adjusted values of *K*_x_ while holding the value of *K*_ox_ under wet conditions constant and back‐calculating *K*_leaf_ (Fig. 4B). Using *K*_x_ in the non‐linear regression equation shown in Fig. 4B, *K*_leaf_ was predicted to be reduced by 50% when *K*_x_ = 11.6% (reduced by 88% from its fully hydrated, ideal value). When *K*_x_ was only 5% of its hydrated value, *K*_leaf_ was predicted to be 30% of its value under wet conditions (Fig. 4B).

### Leaf anatomical variables and correlations with *K*_leaf_

After 14 d without water, leaves of *G. monostachia* showed few external signs of stress, such as wilting or curling. Of the anatomical variables measured in leaf cross‐sections, only the proportional amounts of tissues differed significantly under different conditions of water availability (Table 2; Appendix S2). Specifically, the amount of aerenchyma (AER) as a percentage of both leaf and mesophyll cross‐sectional area was significantly higher under wet conditions than under dry. Rewetting restored the amount of AER relative to the mesophyll but not to the leaf cross‐sectional area (*t* = 1.94, *P* = 0.132). The amount of chlorenchyma relative to the leaf cross‐sectional area was significantly lower under wet and rewetted conditions than under dry (Table 2), whereas the relative amount of hydrenchyma followed an opposite trend, though differences were not significant. The other anatomical traits measured, including leaf thickness, distance from a vein to the epidermis, distance between veins, and vein length per area, did not differ significantly with respect to differences in water availability.

**Table 2:** Leaf anatomical traits of *Guzmania monostachia* under wet, dry, and rewetted conditions. Data are results of one‐way ANOVA (*F* and *P* values) followed by trait means ± SE (different lower case letters indicate a significant difference among treatments; *P* < 0.05, Bonferroni post hoc test; *N* = 6–7 plants for each condition). Abbreviations: AER = aerenchyma, CHL = chlorenchyma, HYD = hydrenchyma, DVE = distance from vein to abaxial epidermis, DIV = distance between the centers of adjacent veins, VLA = vein length per area.

Trait	*F*	*P*	Wet	Dry	Rewetted
**AER/leaf (%)**	**4.75**	**0.022**	**17.4 ± 0.33 a**	**15.0 ± 0.60 b**	**15.8 ± 0.72 b**
**AER/mesophyll (%)**	**12.76**	**< 0.001**	**41.0 ± 0.53 a**	**33.7 ± 1.12 b**	**37.6 ± 1.26 a**
**CHL/leaf (%)**	11.93	**< 0.001**	**25.0 ± 0.46 a**	**29.4 ± 0.78 b**	**26.3 ± 1.74 a**
HYD/leaf (%)	1.90	0.178	57.5 ± 0.64	55.7 ± 0.96	57.9 ± 0.96
Leaf thickness (mm)	3.467	0.227	0.40 ± 0.08	0.38 ± 0.09	0.38 ± 0.11
DVE (μm)	0.294	0.749	164.4 ± 4.5	158.8 ± 5.4	161.6 ± 5.5
DIV (μm)	0.349	0.710	287.9 ± 10.4	277.8 ± 10.5	288.4 ± 9.4
VLA (mm mm^−2^)	0.333	0.721	3.91 ± 0.09	3.80 ± 0.12	3.92 ± 0.12

Note

AER/leaf refers to cross‐sectional area of region as percentage of leaf cross‐sectional area (as shown in Fig. 1A).

Multiple correlation analyses indicated that *K*_leaf_ was significantly and positively correlated with the amount of aerenchyma per leaf cross‐sectional area (AER/lf; Table 3), and negatively correlated with the amount of chlorenchyma per leaf (CHL/lf). There was a strong negative correlation between AER/lf and CHL/lf, indicating that these two traits were not independently related to *K*_leaf_. No other measured anatomical trait was significantly correlated with *K*_leaf_, and the amount of hydrenchyma per leaf cross sectional area and CHL/lf were the only anatomical variables besides AER/lf that were significantly (and negatively) correlated with each other (Table 3).

**Table 3:** Pearson product‐moment correlation coefficient (*r*) between leaf hydraulic conductance (*K*_leaf_) and leaf anatomical variables: aerenchyma (AER/lf), chlorenchyma (CHL/lf), and hydrenchyma (HYD/lf) per leaf cross‐sectional area, leaf thickness (Lf thick.), distance from vein to abaxial epidermis (DVE), distance between the centers of adjacent veins (DIV), vein length per area (VLA). In each column, under the trait are the test statistics *r* and *P*; for each trait, *N* = 19−21.

Leaf trait Test statistic	AER/lf	CHL/lf	HYD/lf	Lf thick.	DVE	VLA	DIV
*K*_leaf_							
* r*	**0.595**	**−0.483**	0.0832	0.0114	0.0811	0.370	0.165
* P*	**0.00719**	**0.0362**	0.735	0.963	0.741	0.119	0.500
AER/lf							
* r*		**−0.457**	−0.245	0.116	0.200	0.122	0.317
* P*		**0.0430**	0.299	0.627	0.398	0.608	0.173
CHL/lf							
* r*			**−0.751**	−0.255	−0.161	−0.119	−0.310
* P*			**0.000136**	0.279	0.499	0.616	0.184
HYD/lf							
* r*				0.195	0.0303	0.0428	0.104
* P*				0.409	0.899	0.858	0.662
Lf thickness							
* r*					0.364	0.155	0.0726
* P*					0.105	0.503	0.754
DVE							
* *r						0.332	0.0589
* *P						0.142	0.800
VLA							
* *r							−0.203
* *P							0.377

**Figure 5:**
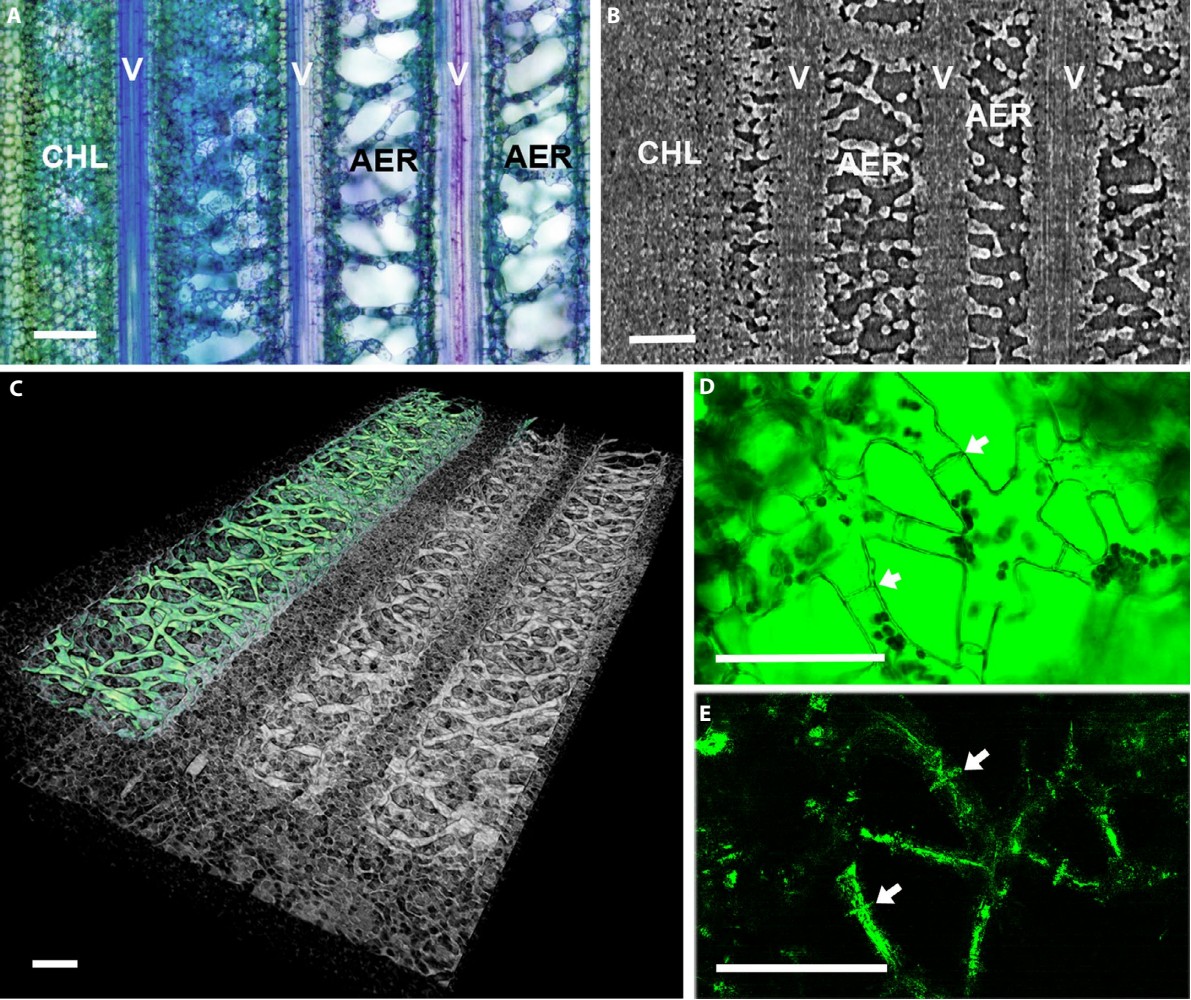
Aerenchyma channels and associated cells in leaves of *Guzmania monostachia* under wet conditions: paradermal sections viewed with (A) a light microscope and (B) microCt‐scanning. CHL, chlorenchyma; V, vein; AER, aerenchyma. (C) 3D representation of three aerenchyma channels; mesophyll strands in one channel are colored in green. (D) Green‐filtered light and (E) fluorescent close‐ups of stellate mesophyll cells in leaf stained with sulforhodamine G (arrows: cell walls). Scale bars: (A–C) 100 μm, (D, E) 50 μm.

Because of the relationship between *K*_leaf_ and AER/lf on a cross‐sectional basis, a 3D analysis of the relative amount of intercellular air space was done using microCT‐scanning. The volume of air space in the regions between the veins was quantified for wet and dry leaves on a per channel basis at several positions along the leaves (channel defined as the region bounded by the upper and lower epidermis, excluding the veins and bundle sheaths on either side). Paradermal images of channels are shown in Figs. 5A‐B, and a 3D image of three aerenchyma channels reconstructed from the microCT‐scanning images is shown in Fig. 5C (mesophyll cells traversing the aerenchmya—also known as stellate mesophyll (Tomlinson, 1969)—are colored green in the left‐hand channel). The total volume of air space for all channels at the midleaf position was 2.2× higher for wet leaves than for dry (*t* = 5.22, *P* < 0.05). However, this difference disappeared when the volume of air space was expressed as a percentage of the channel volume, which for the wet leaf was 23.9 ± 0.4% versus 23.3% ± 1.1% for the dry leaf (Appendix S3; *t* = 0.534, *P* = 0.608). It should be noted that these data are from only two plants per condition, although from multiple positions and channels within each leaf.

Water movement though extravascular tissues was detected by allowing detached leaves to take up the stain sulforhodamine G and viewing cross sections with a confocal microscope (Fig. 5D, E). Stellate mesophyll cells (mesophyll cells with arm‐like projections) of leaves of plants under wet conditions showed stain‐induced fluorescence, primarily in their cell walls (Fig. 5D, E; arrows indicate cell walls), suggesting probable apoplastic transport, whereas the same cell type from leaves under dry conditions showed no such fluorescence (data not shown).

### Aquaporins

Genomic and cDNA sequences from wet, dry, and rewetted glasshouse plants of *G. monostachia*, amplified by gene‐specific primers designed for PIP aquaporins in the closely related bromeliad *Tillandsia ionantha*, yielded a putative amino acid consensus sequence with 93% similarity to *TiPIP2a* of *T. ionantha* (Ohrui et al., 2007), with three putative transmembrane domains indicated by arrow‐headed bars (Appendix S3). When sequences were compared using BLAST, the greatest similarity (87%) was with putative aquaporin *PIP2‐2* in pineapple (*Ananas comosus*), also a member of the Bromeliaceae. Because we cannot distinguish the homolog more precisely, we refer to the gene derived from the single Sanger‐sequenced amplicon as *GmPIP*.

**Figure 6:**
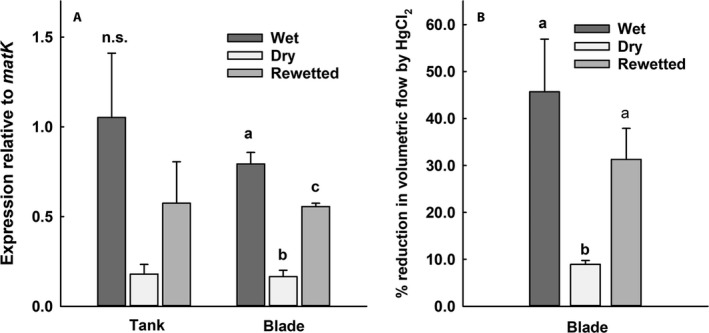
(A) Aquaporin (AQP) mRNA accumulation after 14 d of drying and 4 d of rewetting for field‐collected plants of *Guzmania monostachia* at La Selva. Relative expression data (qRT‐PCR; average ± SE,* N* = 3 biological replicates) are represented on a log 2 scale, *y*‐axis relative to the control. Data were normalized to the reference gene *matK*. Different lower case letters indicate significant differences (*P* < 0.05, one‐way ANOVA). (B) Percentage reduction in *Q*, volumetric water flow calculated as 1 – (*Q* after/*Q* before immersion in 200 μmol HgCl_2_) × 100; different lower case letters indicate significant differences (*P* = 0.038; one‐way ANOVA).

Transcript accumulation of putative aquaporin *GmPIP* was measured in tank and blade regions of leaves under wet, dry, and rewetted conditions in the glasshouse in Los Angeles. As normalized to the reference gene *matK*,* GmPIP* mRNA expression was ca. 5× higher for tank and blade tissue under wet conditions than under dry, and 3× higher for rewetted tissue (Fig. 6A), although differences were significant only for blade tissue (*F*_2, 7_ = 18.96, *P* = 0.001).

The role of aquaporins in regulating *Q*, volumetric water flow, was assessed by comparing leaves measured before and after immersion of the cut end in the inhibitor HgCl_2_ (Fig. 6B). Under wet conditions, the reduction in water flow under the influence of HgCl_2_ was 46%, which was about 5× greater than the reduction for leaves after 14 d of drying (Fig. 6B; *F*_2, 16_ = 4.05, *P* = 0.038). The reduction for leaves after 4 d of rewetting was 31%, and the value did not differ significantly from that under wet or dry conditions.

### Cuticular conductance

**Figure 7:**
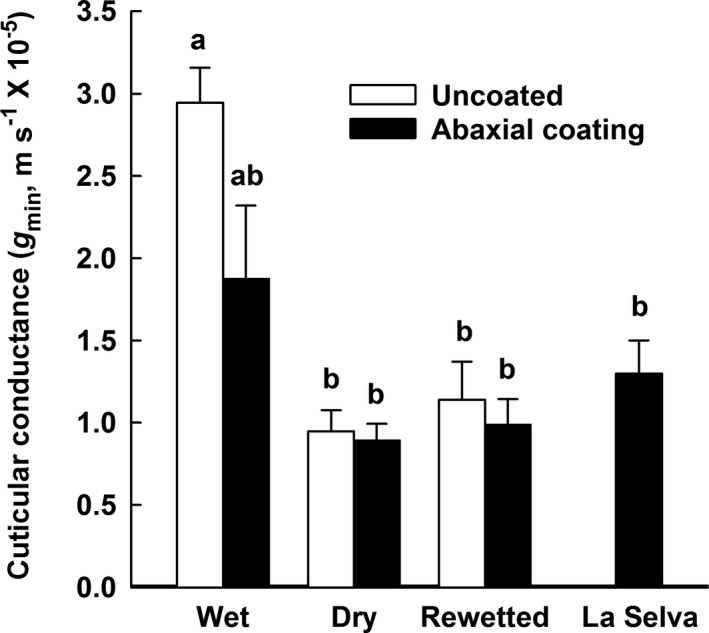
Cuticular conductance (*g*_min_) for leaves of *Guzmania monostachia* in Los Angeles and La Selva. For half of the leaves, silicon grease was applied to the abaxial surface to occlude stomates, and *g*_min_ was calculated from leaf mass loss, leaf temperature, and ambient relative humidity; *N* = 5 leaves for each treatment; different lower case letters indicate significant differences (*P* < 0.001; one‐way ANOVA).

Under wet conditions in the glasshouse, cuticular conductance (*g*_min_) for leaves with their abaxial (lower) surfaces uncoated was about 2–3× higher than *g*_min_ for coated and uncoated leaves under dry and rewetted conditions, as well as for coated leaves at La Selva (Fig. 7; *F*_4, 20_ = 9.201, *P* < 0.001). The purpose of the coating was to occlude stomates, which occur only on the abaxial side of *G. monostachia* leaves (North et al., 2013). Abaxial coating did not reduce *g*_min_ under dry and rewetted conditions and had a slight but nonsignificant effect for leaves under wet conditions (*t* = 2.84, *P* = 0.10).

## DISCUSSION

The variations measured in leaf hydraulic conductance (*K*_leaf_) for *Guzmania monostachia* reflected a reduction in leaf water loss under dry conditions and resilience in water uptake and transport after rewetting. Although the whole‐leaf staining method indicated a significant increase in xylem embolism and an inferred decrease in xylem conductance (*K*_x_) during drought, decreases in hydraulic conductance outside the xylem (*K*_ox_) were more substantial. Hydraulic changes were accompanied, if not fully explained, by changes in leaf anatomy, cuticular conductance (*g*_min_), and aquaporin expression. A 14‐d period with no external supply of water decreased *K*_leaf_ by half, yet leaves of *G. monostachia* proved resilient during both drying and rewetting, showing few obvious signs of water stress in either field or glasshouse settings. Measurements of *K*_leaf_ made after 30 d of drought (*N* = 3) showed no further reduction, supporting the conclusion that the wide range and broad environmental tolerance of this tank bromeliad may be due to its flexible yet conservative use of water, paralleling its ability to switch between C_3_ and CAM photosynthesis (Maxwell et al., 1994).

### Leaf hydraulic conductance and its components

In terms aligned with other studies on leaf hydraulic conductance (Scoffoni and Sack, 2017), the leaf water potential (*Ψ*_leaf_) at which *K*_leaf_ for *G. monostachia* was halved, or its *P*_50_, was –0.8 MPa after 14 d of drought imposed in the greenhouse (Fig. 2). The same *Ψ*_leaf_ was recorded for plants of *G. monostachia* at the height of the dry season in a moist tropical forest in Panama (Zotz and Andrade, 1998). Moreover, despite a decrease of *Ψ*_leaf_ to –0.99 MPa for 3 plants after 30 d with no water in the glasshouse, no further decline in *K*_leaf_ occurred. For other epiphytic bromeliads, *P*_50_ was about the same as that measured for the C_3_ tank bromeliad *G. lingulata*, more negative than for a CAM atmospheric species, and less negative than for a CAM tank bromeliad (Males and Griffiths, 2018), which is in keeping with the status of *G. monostachia* as a C_3_–CAM intermediate (Griffiths et al., 1986). The semi‐succulent leaf bases that form the tank in this species (North et al., 2013) could have supplied water to the leaf blades, thereby helping to prevent large decreases in *Ψ*_leaf_ and *K*_leaf_ during drought.

The refilling of tanks of *G. monostachia* after drought in the field and in the glasshouse led to increases in *K*_leaf_ within 4 d; for glasshouse plants, *K*_leaf_ for rewetted plants equaled or exceeded its value under wet conditions. Recovery in *K*_leaf_ after dehydration has been observed for other species within 1 h of rehydration, yet such measurements have usually been made on detached leaves rehydrated directly in water (Trifilò et al., 2003; Scoffoni et al., 2012). Recovery in *K*_leaf_ following rewetting of whole plants after drought often takes longer than that and may depend on the level of water stress (Blackman et al., 2009). The ability of leaves of *G. monostachia* to recover over 80% of their relative water content after tissue water loss of 90% (Zotz and Andrade, 1998) suggests strong resilience for this species with respect to *K*_leaf_, even after a more prolonged drought than that imposed in this study.

Whole‐leaf staining with acid fuchsin (Fig. 3) appeared to be a reliable method to detect nonconducting veins, and by inference embolized veins, for a number of reasons. First, the stain traveled to leaf apices within 2 h, unlike basic fuchsin, phloxine, and safranin, which moved more slowly and more unevenly, presumably due to their more basic pH; second, the stain did not appear to diffuse beyond bundle sheaths into surrounding tissues. An objection to the staining method is that dye movement in drought‐exposed leaves could be limited by stomatal closure (Scoffoni and Sack, 2017); however, leaves in this study were exposed to the same conditions as those used in measuring *K*_leaf_, when stomates were presumably open. The number of stained veins decreased by 17% from wet to dry conditions, suggesting a relatively modest level of embolism, which was reversed by rewetting. After 30 d without water, stain uptake by leaves indicated that about 40% of the veins were nonconducting.

Changes in the relative contributions of pathways inside and outside the xylem were analyzed using a leaky cable model, perhaps most easily visualized in terms of relative resistances (Fig. 4A). Xylem resistance, *R*_x_, was lower than outside‐xylem resistance, *R*_ox_, under all conditions, indicating that the extravascular pathway was the more limiting, particularly during drought. Even though embolism increased *R*_x_ to about 40% of the summed resistances under dry conditions, in terms of impact on *K*_leaf_, a 50% reduction in *K*_x_ was predicted to decrease *K*_leaf_ by only 15%, and a 50% reduction in *K*_leaf_ would require an 88% reduction in *K*_x_ (Fig. 4B). Values for *K*_x_ were derived from the Hagen–Poiseuille equation and may thus overestimate actual conductance; however, combined modeling and experimental studies of component resistances in flow through the tracheids of the whisk fern *Psilotum nudum* (Schulte et al., 1987) and the fern *Pteris vittata* (Calkin et al., 1986) showed that most of the hydraulic resistance was due to the lumen, not the pit membranes, of narrow tracheids similar in diameter to those of *G. monostachia*; moreover, *K*_x_ for small‐diameter conduits was better approximated by the Hagen–Poiseuille equation than was *K*_x_ for larger conduits. Still, estimates of *K*_x_ based solely on tracheid diameters do not take into account the connectivity of the vascular pathway, which is likely to become more fragmented and tortuous when disrupted by embolism (Jacobsen and Pratt, 2018; Mrad et al., 2018). Thus, although drought‐induced decreases in *K*_x_ were modeled to have less of an effect on *K*_leaf_ in *G. monostachia* than were changes outside the xylem, using measured values of *K*_x_ in the model may have yielded different results.

### Anatomical changes during and after drought

Few of the anatomical variables measured in this study were influenced measurably by drought or rewetting. Because of differences in leaf thickness and number of veins from leaf to leaf, tissue area was expressed as a percentage of overall leaf or mesophyll area, which might obscure significant differences caused by shrinkage and swelling in response to drying and rewetting. However, leaves showed few gross changes during 14 d of drought aside from slight browning of leaf tips; little wilting or turgor loss was observed. In any case, only the relative proportions of tissues within leaves varied, and seemingly in counterintuitive ways. The percentage of chorenchyma per leaf increased during drought, perhaps reflecting water imported from storage in the hydrenchyma, which showed a slight but insignificant decrease. Although not measured, water could also have been imported into the photosynthetically active chlorenchyma from storage in leaf bases in the tank region, similar to water transfer from tissue to tissue in leaves of CAM plants during drought (Nobel et al., 1994; Nowak and Martin, 1997). The changes in chlorenchyma per leaf area were negatively correlated with changes in *K*_leaf_ (Table 3), possibly signifying an increase in path length for water from the xylem to the surface of the leaf. However, in *G. monostachia* most of the chlorenchyma is adaxial, or above the plane of the veins, and stomates are strictly abaxial (Fig. 1), suggesting that most water does not travel directly through the chlorenchyma. Instead, the path for water movement outside the xylem traverses the aerenchyma.

Aerenchyma as a percentage of leaf and mesophyll cross‐sectional areas decreased during drying (Table 1). As defined here, aerenchyma (Figs. 1, 5) includes air lacunae and scattered strands of chloroplast‐containing cells (shown in Fig. 5C–E) known as stellate mesophyll (Tomlinson, 1969) and is not synonymous with intercellular air spaces. The strongest correlation between *K*_leaf_ and any anatomical variable was with aerenchyma/leaf and aerenchyma/mesophyll (Table 3), perhaps because both decreased significantly during drought. A causal connection at first seems unlikely, since an increase in aerenchyma would lengthen the path for water to travel between veins and stomates. Air lacunae and intercellular air spaces can be paths for water movement in the vapor phase, however, with conductances determined by temperature gradients (Rockwell et al., 2014; Buckley, 2015), which could be larger for leaves with higher transpiration rates under wet conditions than during drought. In addition, as indicated by the tracer dye sulforhodamine G, water appeared to move outside the xylem preferentially through stellate mesophyll cells under wet but not dry conditions. Connections between the elongate arms of stellate mesophyll cells could decrease during the shrinkage of aerenchyma under dry conditions, thereby reducing a possible role for these cells in transporting water between veins.

Per volume of leaf (excluding veins), microCT scans revealed similar percentages of air space relative to other leaf tissues under wet and dry conditions, about 23% (Fig. 5B, C), although the total volume of air space was twice as high for wet leaves as for dry. During drying, the scaffolding provided by the stellate mesophyll cells that make up the aerenchyma may have been disrupted, resulting in a greater decrease in air space than in the more closely packed tissues of the mesophyll and hydrenchyma. MicroCT scans indicated approximately an 8% decrease in leaf thickness during drying (data not shown), consonant with the decrease in aerenchyma seen in the 2D cross sections. Thus, both 2D and 3D imaging suggest that extravascular pathways for water flow through the mesophyll were compromised during drying.

### Aquaporin expression and inhibition

Primers based on plasma membrane integral protein (*PIP*) genes previously identified in a closely related bromeliad, *Tillandisa ionantha* (Ohrui et al., 2007), were used to analyze expression of an aquaporin tentatively identified in this study as *GmPIP*. Similar to several other studies that have examined aquaporin expression in leaves in response to drying and rewetting (Laur and Hacke, 2014; Vitali et al., 2016; Shelden et al., 2017; Zupin et al., 2017), transcript accumulation of *GmPIP* was strongly reduced in blade tissue of leaves of *G. monostachia* after 14 d of drought and was partially restored after 4 d of rewetting (Fig. 6A). Although specific homologs were not identified, *GmPIP* is most closely related to *PIP2‐2* in pineapple. It is likely that *G. monostachia* has many aquaporins (e.g., pineapple has 23; Bezerra‐Neto et al., 2019), many with partially redundant functions or varying patterns of expression. Nevertheless, expression levels of *GmPIP* were congruent with drought‐induced changes in *K*_leaf_.

The 5‐fold decrease in *GmPIP* expression was echoed in the 5‐fold reduction in the inhibitory effect of HgCl_2_ on water flow through the leaf; i.e., the inhibitor reduced flow by about 45% under wet conditions and by only about 9% after 14 d of drying, suggesting that aquaporins had already been downregulated. As was the case with aquaporin expression, rewetting did not fully restore aquaporins to their pre‐drying status, as inferred from the 31% reduction in water flow (Fig. 6B). While the identification of specific aquaporin isoforms and other aspects of their involvement in water movement, such as the extent of phosphorylation, was beyond the scope of this study, the congruence between patterns of expression and inhibition strongly suggests that aquaporins were involved in regulating drought‐induced changes in water movement across leaves of *G. monostachia*. Moreover, despite a decline, leaf water potential (*Ψ*_leaf_) of *G. monostachia* did not decrease below –0.8 MPa during 14 d of drought and was no lower than –1.0 MPa after 30 d with no water, possibly implicating aquaporins in helping the leaves of *G. monostachia* to avoid hydraulic risk (Moshelion et al., 2015).

### Cuticular conductance

The last step for water exiting a leaf is across the epidermis, through either stomates or epidermal cells themselves. When stomates are closed or minimally open, water movement through the leaf epidermis is determined by cuticular conductance (*g*_min_), which tends to be comparatively low for bromeliads (Benzing and Burt, 1970; Kerstiens, 1996) and even lower for tropical epiphytes from other families (Helbsing et al., 2000). Values of *g*_min_ for *G. monostachia* (Fig. 7) were comparable to the mean for 20 bromeliads, both terrestrial and epiphytic, and about 10× higher than that measured for 10 tropical epiphytes (Helbsing et al., 2000); the methods used in the latter study (isolated cuticles rather than whole leaves as in this study) may account for some of these differences. Under dry conditions in the glasshouse, *g*_min_ was about 50% of that under wet conditions whether or not the stomates were occluded, and similar to values of *g*_min_ measured for desert species such as grasses (Smith et al., 2006) and desert green‐stem perennials (Ávila‐Lovera et al., 2017). The decrease in *g*_min_ for plants of *G. monostachia* in the glasshouse was not reversed by rewetting. Presumably, the leaves measured on plants at La Selva had gone through several cycles of drying and rewetting, suggesting that cuticles may have undergone irreversible change during drought, as has been reported for wheat leaves (Bi et al., 2017). Exposure to drought may have restricted the final path for water loss for leaves of *G. monostachia*, a path that is particularly important when stomates are closed during drought.

## CONCLUSIONS

Leaf hydraulic conductance (*K*_leaf_) for *G. monostachia* was halved during 2 weeks under dry conditions and was fully restored within 4 days of rewetting. Measured and modeled changes inside (*K*_x_) and outside (*K*_ox_) the xylem pointed to a greater role for *K*_ox_ in determining rates of water movement through the leaf. In particular, changes in the amount of aerenchyma, aquaporin involvement, and cuticular conductance, all of which occurred outside the xylem, paralleled the decrease in *K*_leaf_. With the exception of cuticular conductance, drought‐induced decreases in key traits associated with *K*_ox_ were largely reversed by refilling the tanks, as was the decrease in *K*_x_ due to embolism. Thus, water‐conducting pathways in leaves of *G. monostachia* proved resilient when exposed to a 14‐d drought similar in duration to those expected in its current range, with additional measurements suggesting that a 30 d dry period was also tolerable. The hydraulic resilience of this species should work in concert with the flexibility of its C_3_–CAM photosynthetic pathway to help maintain its broad distribution despite increasing drought. However, it is important to note that all measurements in this study were made under moderate temperatures, probably unlike those that *G. monostachia* and other tropical epiphytes will face in the future.

## AUTHOR CONTRIBUTIONS

G.N. organized data acquisition, performed most of the data analysis, and did most of the writing. E.B. acquired and analyzed most of the gene expression data and contributed to the writing. M.B. and M.G. acquired most of the field data and laboratory data on hydraulics. A.R. acquired and analyzed the MicroCT data with funding and support from C.B., and both contributed to the writing. T.K., E.W., and V.F. acquired data in the field and laboratory and helped with data analysis.

## Supporting information


**APPENDIX S1.** Whole‐leaf sections of *G. monostachia* showing uptake of different stains used to detect embolism.

**APPENDIX S2.** Fresh and fixed cross sections of leaves of *G. monostachia* under wet, dry, and rewetted conditions.

**APPENDIX S3.** Aquaporin **(**PIP1) alignment to previously identified PIPs of *Tillandsia ionantha* and *Ananas comosus*.

## Data Availability

Data and additional figures are available at the digital repository FigShare (https://figshare.com/s/e4399f986365b4445161).
