# Supplementary material for: Hydraulic conductance, resistance, and resilience: how leaves of a tropical epiphyte respond to drought
Source: Am J Bot. 2019 Jul 11;106(7):943–57. doi: 10.1002/ajb2.1323 (PMC6852343; doi:10.1002/ajb2.1323)

**Appendix S1**. Whole-leaf sections of *G. monostachia* after leaves were allowed to take up different stains for 2 h (A) 0.2% acid fuchsin, (B), 0.2% basic fuchsin, (C) 0.1 % phloxine, and (D) 0.2% safranin O, all viewed under a microscope at 40×. Scale bar = 150 µm.


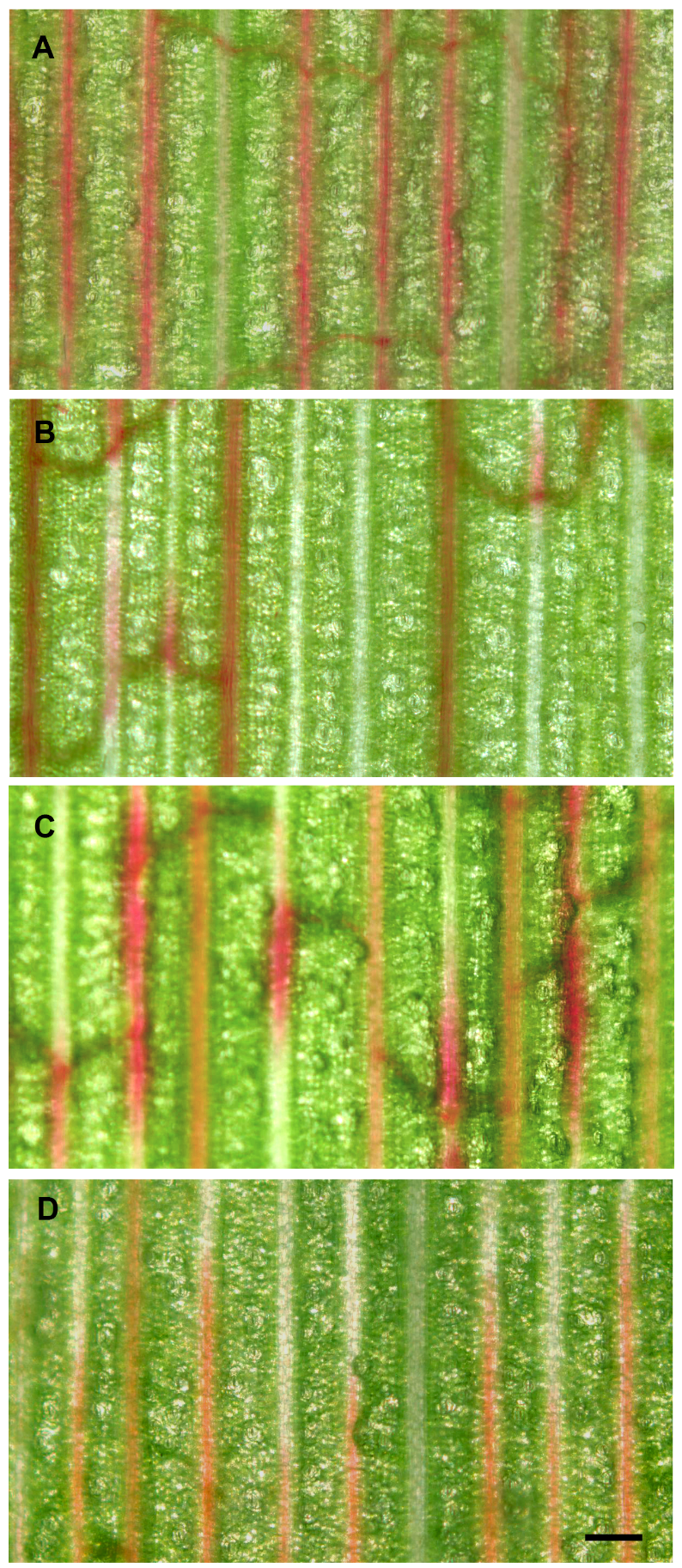

Supplement: Supplementary file 1 — APPENDIX S1. Whole‐leaf sections of G. monostachia showing uptake of different stains used to detect embolism. [file AJB2-106-943-s001.docx]
