# Supplementary material for: Hydraulic conductance, resistance, and resilience: how leaves of a tropical epiphyte respond to drought
Source: Am J Bot. 2019 Jul 11;106(7):943–57. doi: 10.1002/ajb2.1323 (PMC6852343; doi:10.1002/ajb2.1323)

**Appendix S2:** Cross sections of leaves of *Guzmania monostachia* under (A, B) wet, (C, D) dry and (E, F) rewetted conditions, either freshly cut (Unfixed, A, C, E) or after leaves were fixed in FAA (Fixed, B, D, F). All leaves were hand-sectioned with a razor blade, stained with toluidine blue O, and photographed at 100×; scale bar = 100 µm.

**Unfixed Fixed**


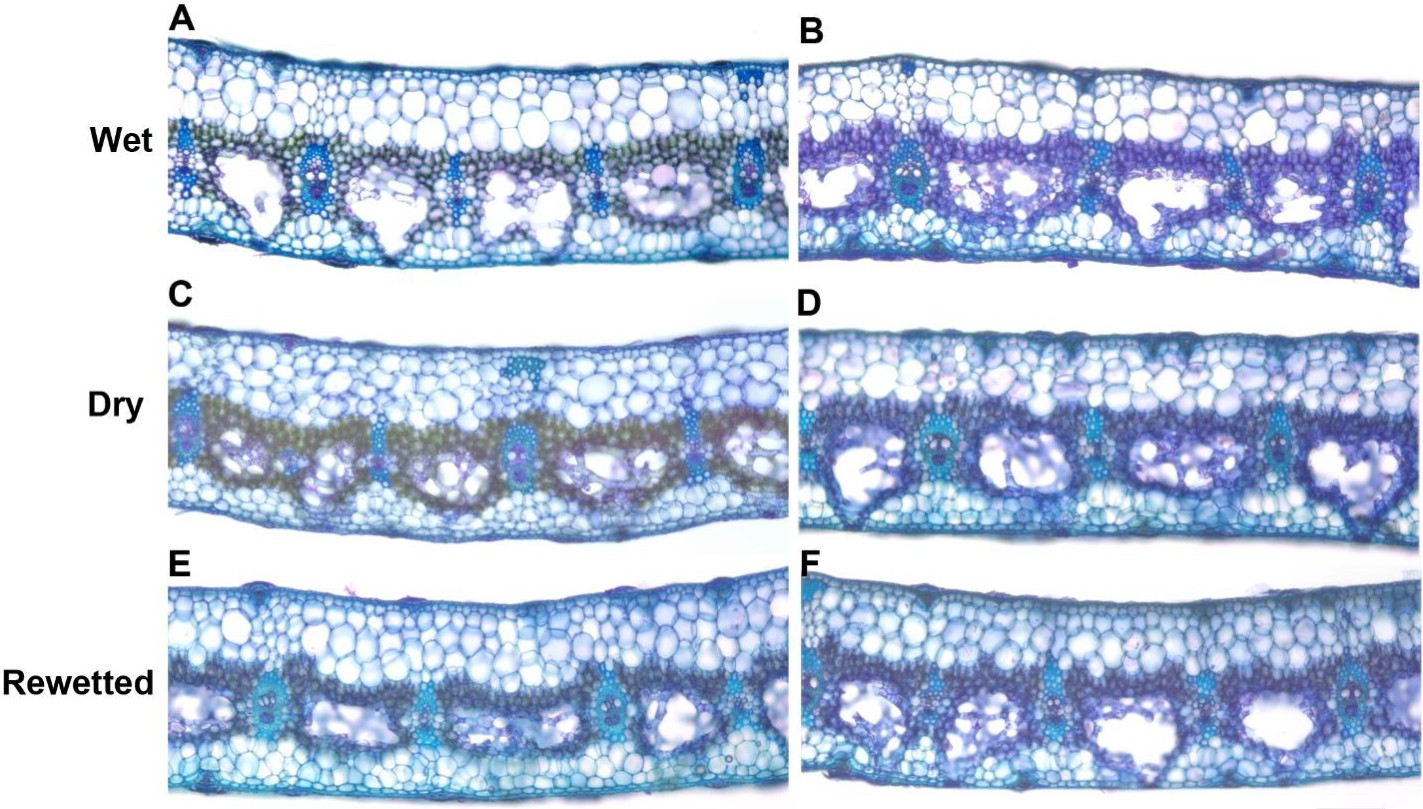

Supplement: Supplementary file 2 — APPENDIX S2. Fresh and fixed cross sections of leaves of G. monostachia under wet, dry, and rewetted conditions. [file AJB2-106-943-s002.docx]
