# Supplementary material for: Hydraulic conductance, resistance, and resilience: how leaves of a tropical epiphyte respond to drought
Source: Am J Bot. 2019 Jul 11;106(7):943–57. doi: 10.1002/ajb2.1323 (PMC6852343; doi:10.1002/ajb2.1323)

**Appendix S3:** Amino acid sequence alignments of aquaporin **(**PIP1) from *Guzmania monostachia*, *Tillandsia ionantha* ([Ohrui et al., 2007](#_ENREF_41)) and *Ananas comosus* (pineapple) (NCBI). Shading indicates degree of conservation among sequences (black box, white text = 100%; gray box, white text = 80%; gray box, black text = 60%). Sequence for *G. monostachia* was deduced from genomic and cDNA sequence data.


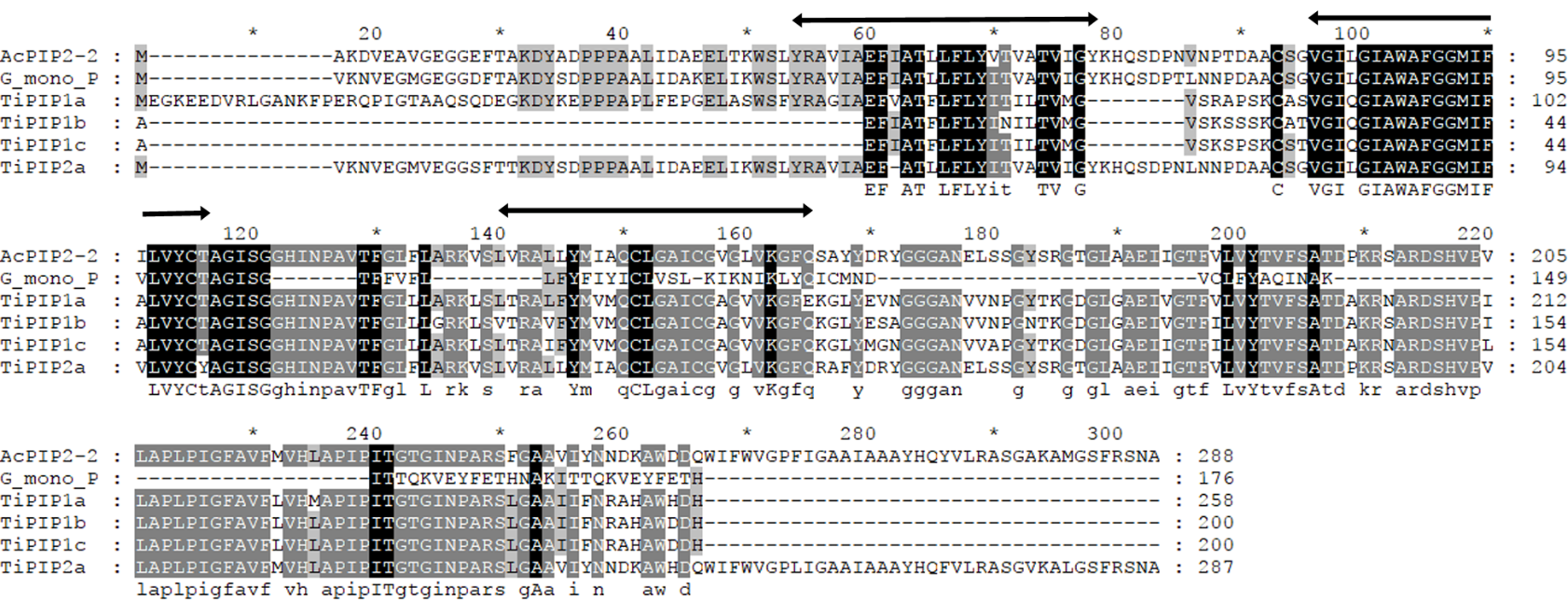

Supplement: Supplementary file 3 — APPENDIX S3. Aquaporin (PIP1) alignment to previously identified PIPs of Tillandsia ionantha and Ananas comosus. [file AJB2-106-943-s003.docx]
